# Parents’ underestimation of their child’s weight status. Moderating factors and change over time: A cross-sectional study

**DOI:** 10.1371/journal.pone.0227761

**Published:** 2020-01-16

**Authors:** Emilie L. M. Ruiter, Jenneke J. E. H. Saat, Gerard R. M. Molleman, Gerdine A. J. Fransen, Koos van der Velden, Cornelia H. M. van Jaarsveld, Rutger C. M. E. Engels, Willem J. J. Assendelft

**Affiliations:** 1 Academic Collaborative Center AMPHI, Integrated Health Policy, Department of Primary and Community Care, ELG, Radboud university medical center, HB Nijmegen, the Netherlands; 2 Department of Primary and Community Care, ELG, Radboud university medical center, HB Nijmegen, the Netherlands; 3 Erasmus University Rotterdam, DR Rotterdam, the Netherlands; University of Bern, SWITZERLAND

## Abstract

**Background:**

Parents’ underestimation of their child’s weight status can hinder active participation in overweight prevention programs. We examined the level of agreement between the parents’ perception of their child’s weight status and the child’s actual weight status, moderating factors, and change over time.

**Methods:**

This cross-sectional study used data collected in 2009 (n = 8105), 2013 (n = 8844) and 2017 (n = 11,022) from a community-based survey conducted among parents of children age 2–12 years in the Netherlands. Parents classified their perception of their child’s weight status on a 5-point Likert scale. In 2009 and 2013, the child’s BMI was calculated from self-reported data by parents. The level of agreement between the parent’s perception of the weight status and the actual weight status was examined using Cohen’s kappa. The role of demographic factors on parents’ perception were examined using logistic regression.

**Results:**

In 2009, 2013 and 2017, 6%, 6% and 5% of the parents, respectively, classified their child as heavy/extremely heavy. In 2009 and 2013, 64.7% and 61.0% of parents, respectively, underestimated the weight status of their overweight child. This was even higher among parents of obese children. Overall, the agreement between the parents’ perception and the actual weight status improved from 2009 (kappa = 0.38) to 2013 (kappa = 0.43) (p<0.05), but remained unsatisfactory. The parents’ underestimation of their child’s overweight/obesity status was associated with the child’s age in 2009 and 2013 (2–7 years; OR: 0.18), the child’s gender in 2009 (male; OR: 0.55), and the parents’ education level in 2009 (middle and high education; OR: 0.56 and 0.44 respectively).

**Conclusions:**

Parents’ underestimation of their child’s weight status remains alarmingly high, particularly among parents of young, obese children. This underestimation is a barrier to preventing childhood overweight/obesity. Healthcare professionals should take this underestimation into consideration and should actively encourage parents to take steps to prevent overweight/obesity in their children.

## Introduction

In children, overweight and especially obesity are serious health issues [1, 2]. Preventing the development of both overweight and obesity is important, particularly given the complexity associated with treating these conditions [3] and their far-reaching, long-term consequences [4–8]. A child who is overweight or obese is more likely to remain overweight or obese in adulthood [9, 10], is more likely to suffer from detrimental social consequences such as depression, low self-esteem, and social stigma [4–6], and is more likely to develop chronic diseases later in life, including type 2 diabetes and cancer [7, 8].

Public health policy-makers and clinicians agree that there is a clear need to implement population-wide prevention programs in early childhood in order to curb the obesity epidemic [11] and to identify and treat both overweight/obese children and children who are at risk for becoming overweight or obese [12, 13]. Parents can clearly influence their child’s development of healthy energy balance‒related behaviors and are important role models both in terms of promoting these healthy behaviors in their child’s micro-environment and in terms of dealing with numerous environmental obesogenic factors [14, 15]. Thus, parents’ active participation in intervention programs is an important component in both prevention and treatment of childhood overweight/obesity [16, 17], and parents’ accurate awareness of (the impact of) their child's weight status is of the utmost importance for parents to be willing to participate in such intervention programs. Moreover, evidence suggests that parents who feel that their child’s weight is a health problem are significantly more likely to introduce changes in their child’s lifestyle compared to parents who do not recognize this problem [18]. In addition, results from international studies show that more than half of all parents of overweight/obese children 2–12 years of age either underestimate their child’s weight status or are not concerned regarding the risks associated with childhood overweight/obesity [16, 17, 19–21]. This parental underestimation of the child’s weight status is alarming and can have several important consequences, including: *i*) hindering the parents’ motivation to address the problem [18, 22]; *ii*) hindering the parents’ active participation in intervention programs as well as participation of overweight/obese children; *iii*) causing overweight children to regard their weight status as “normal” and therefore continue to engage in obesogenic behaviors; and *iv*) increasing the risk of non-overweight children becoming overweight in the future [23].

Therefore, empirical data about parents’ perception of their child’s weight status is needed in order to help healthcare professionals organize and adapt activities and programs that promote healthy weight status among children. When parents’ perceptions regarding their child’s weight status are adjusted by a healthcare professional, the parents are more likely to move from the precontemplation stage of change to the preparation or action stage of change toward helping their overweight or at-risk child make the appropriate changes to his/her lifestyle [18]. International studies, a meta analysis and systematic reviews revealed that the child’s age, gender, ethnicity, and weight status, as well as the parents’ education level, can affect the parents’ perceptions of the child’s weight status [16, 17, 20, 22, 24, 25]. However, these findings are generally inconsistent. An explanation for the discrepancies between studies may be due to differences in the age-range of included children, the BMI cut-off criteria to define overweight, the size of the study population, and choices of conducted subgroup analyses [16, 17, 20, 24]. Moreover, insight into the change of the parents’ perception of their child’s weight status over time is missing. With the exception of age, no Dutch studies have found any clear moderating factors [22, 24–26]. Importantly, no Dutch study to date has examined parents’ perception of their child’s weight status with an age range of children 2 to 12 years of age. Knowledge of this missing information is useful to understand the motivation of parents, which could help individual professionals, inspire interventions to prevent childhood overweight/obesity and support policy making.

Therefore, the aim of this study was to examine the level of agreement between the parents’ perception and the child’s actual weight status (based on the child’s BMI calculated from self-reported height and weight by parents), the factors that moderate this perception, and changes over time (2009–2017) in a large Dutch cohort of parents of children 2–12 years of age.

## Methods

### Study design and database

For our repeated measure cross-sectional, survey-based study, we had access to the Child Monitor, a database managed by the Municipal Health Services Department of the Gelderland-South region in the Netherlands. This database is part of a cross-sectional, survey-based study that was conducted in 2009, 2013 and 2017. The purpose of which was to gain insight into the population’s health, lifestyle, and well-being of children age 6 months to 12 years living in the Gelderland-South region of the Netherlands [27]. The survey was conducted as part of a monitoring cycle performed every four years as required by the Dutch Public Health Act. Because these data are routinely collected and are anonymous, and because participation in the survey was voluntary, an active informed consent was not required for this survey. The research population that was asked to complete the Child Monitor questionnaire was well informed about the aim of the Child Monitor. The design and method (data collection) of the Child Monitor complies with the legal provisions of the Personal Data Protection Act and the General Data Protection Regulation in the Netherlands. It also complies with the protocols of the Municipal Health Service that are part of the Dutch Harmonization of Quality Assessment in the Healthcare Sector (HKZ) system that is periodically tested externally. We received approval from the Director of the medical office of the Municipal Health Services Department of the Gelderland-South region in the Netherlands to use the Child Monitor database for our analyses and to report the results. This director is formally responsible for conducting of the Child Monitor. For the purpose of our study, we used the data collected from parents with a child 2–12 years of age at the time of the survey, as the international threshold data for childhood overweight as recommended by the International Obesity Task Force (IOTF) [28], see S1 Appendix, are only available for children age 2 years and older.

### Study population

In 2009, a total of 15,991 parents/caregivers in the Gelderland-South region of the Netherlands with a child between the ages of 6 months and 12 years were randomly selected and invited to complete a validated questionnaire (either digitally or on paper). A total of 9,796 parents (61%) completed the questionnaire, and 8,105 of these questionnaires were about children between 2–12 years of age at the time the questionnaire was completed. In 2013, the same procedure was followed. From 22,601 randomly selected parents/caregivers, 10,161 (45%) completed the questionnaire; 8,844 of the children were 2–12 years of age at the time the questionnaire was completed. In 2017, a different procedure was used for recruiting. In 2017, only parents with a child between the ages of 6 months and 4 years were randomly selected as described above for 2009 and 2013. Of the 10,142 randomly selected parents/caregivers in the Gelderland-South region with a child between the ages of 6 months and 4 years, at total of 4,342 parents (43%) completed the questionnaire, of which 2,719 children were 2–4 years of age at the time the questionnaire was completed. Furthermore, in 2017 all 31,168 parents/caregivers of children 4–12 years of age were approached via the child’s primary school; 229 of the 246 primary schools (93%) agreed to cooperate. The parents of the children enrolled at these 229 schools received a link to the Child Monitor questionnaire via the school’s newsletter or via an e-mail from the school. From 31,168 approached parents/caregivers a total of 8,303 (27%) completed the questionnaire. Thus, in 2017 a total of 11,022 questionnaires (2,719 + 8,303) were regarding children who were 2–12 years of age at the time the questionnaire was completed.

To answer research questions at the district level, all sets of samples (i.e., from 2009, 2013 and 2017) were increased at the district level, with specific defined focus areas (unequal sampling), which is also referred as a complex sample design. In these sample sets, ethnic minorities and low socio-economic status (SES) respondents were deliberately oversampled.

### Measures

#### Socio-demographic characteristics

The following demographic data were obtained from parents via the Child Monitor questionnaire: the child’s age, gender, and ethnicity, and the education level of both parents (as an indicator of SES). The child’s age was dichotomized into the following two groups: 2–7 years and 8–12 years. Ethnic background was determined by asking the country of birth of the child and of both parents. The parents were able to choose from the following six categories: “The Netherlands”, “Turkey”, “Morocco”, “Surinam”, “Netherlands Antilles”, or “Other country”. The results were classified according to standard procedures established by Statistics Netherlands [29]: “Caucasian” (native-born and western migration background) and “non-Caucasian” (non-western migration background). The parents’ level of education was based on the highest level achieved and was classified as follows in accordance with international classification systems [30]: “low” (lower general secondary education, lower vocational training, or primary school), “middle” (intermediate vocational training, higher general secondary education, or pre-university education), or “high” (higher vocational training or university education). If the educational level of both parents differed from each other, then the following criteria were used: If the parents’ level of education of one parent is low and the other is middle, then the education level of both parents is low; If parents’ level of education of one parent is low and the other is high, then the education level of both parents is middle; If the parents’ level of education of one parent is middle and the other is high, then the education level of both parents is middle.

#### Parents’ perception of their child’s weight status

Parents’ perception of their child’s weight status was determined by asking parents the following question: *“What do you think of* your child’s *weight*?*”*. The five possible answers were as follows: ‘‘extremely low”, “low”, “normal”, “heavy”, and “extremely heavy”. Parents were instructed to select the answer that they felt best suited their child’s weight status. The answers were then classified as follows: “not heavy” (extremely low, low and normal), “heavy”, or “extremely heavy”.

#### Weight and height status

The child’s body weight and height were determined by asking the following questions: “*What is the current weight of your child in kg (without clothing)*?” and “*What is the current height of your child in cm (without shoes)*?”. Only the Child Monitor questionnaires of 2009 and 2013 included these two questions.

### Data analysis

First, we calculated each child’s body mass index (BMI) based on the child’s weight and height reported by the parents in 2009 and 2013. Each child’s BMI was then converted to weight status, which was classified into three categories (“not overweight”, “overweight”, or “obese”) based on international cut-off values for childhood overweight as recommended by the IOTF [28].

Next, complex samples statistics were used to calculate the mean values and frequencies from the 2009, 2013, and 2017 databases of the dichotomized demographic variables, the parents’ perceptions of their child’s weight status, and the child’s actual weight status based on BMI. To examine if the differences in the distribution of these variables between the survey years were significant we used Chi-square tests. Because the Child Monitor survey utilized a complex sample design, a sample weight was assigned to each person sampled and therefore complex samples statistics were used to produce an unbiased regional estimate [27, 31].

Because the Child Monitor questionnaire did not contain any questions regarding the child’s height or weight, we were unable to determine the weight status of the children in the 2017 dataset.

Next, we assessed agreement between the parents’ perception of their child’s weight status and the actual weight status. To quantify the parents’ awareness of their child’s weight status, we calculated percent agreement. This percentage provides a measure of the sensitivity with which parents correctly identified their child as being overweight/obese. In addition, we quantified the reliability of this percent agreement using Cohen’s kappa from complex samples analysis with linear weighting. Cohen’s kappa is a statistic that corrects for agreement by chance. The value for Cohen’s kappa ranges from 0 to 1 as follows: 0, no agreement; <0.2, poor agreement; 0.2 to <0.4, moderate agreement; 0.4 to <0.6, reasonable agreement; 0.6 to <0.8, good agreement; 0.8 to <1, very good agreement; and 1, full agreement [32].

Next, to determine whether the agreement between the parents’ perception of their child’s weight status and the child’s actual weight status changed over time, we compared the kappa value calculated for the 2009 dataset with the kappa value calculated from the 2013 dataset using the two-sample z-test. Logistic regression analyses were then performed for both the 2009 and 2013 datasets in order to establish two models. In both models the parents’ perception of their child’s weight status (not heavy vs heavy/extremely heavy) was the outcome. Model 1 examined the role of the child’s actual weight status (not overweight vs. overweight/obese) on the parents’ perception of their child’s weight status. Also, Model 1 examined the role of the following demographic factors on the parents’ perception of their child’s weight status: the child’s age (2–7 years vs. 8–12 years); the child’s gender; the child’s ethnicity (Caucasian vs. non-Caucasian); and the parents’ educational level (low vs. middle/high). Model 1 included the child’s actual weight status as a variable, and each demographic factor was entered separately. In a separate analysis, we examined possible interactions between these factors. We used interaction terms to test whether each of these demographic factors modified the effect of parents’ perception of their child’s weight status, because these variables are related to the child’s weight status. Model 2 (fully adjusted) included the child’s actual weight status, and all four demographic factors were entered simultaneously.

Lastly, as subgroup analysis, logistic regression analyses were performed for both the 2009 and 2013 datasets in order to examine the putative role of demographic factors on the parents’ perception of their child’s weight status of parents of overweight/obese children.

Statistical calculations were performed using IBM SPSS Complex Samples Statistics version 20, with the exception of Cohen’s kappa complex samples analysis, which was performed using the online program VassarStats (http://vassarstats.net/) [33].

## Results

### Socio-demographic characteristics

The socio-demographic characteristics of the children and parents, the parents’ perceptions of their child’s weight status, the children’s weight status based on the children’s BMI, and the differences in the distribution of these variables between the survey years are summarized in Table 1. Apart from gender all variables had significant different distributions between the survey years. With increasing years the database consisted of slightly fewer younger children, fewer non-Caucasian children, fewer parents with a low level of education, and more parents with a high level of education, and more parents’ with the perception of their child’s weight status as not heavy.

**Table 1:** Socio-demographic characteristics of the study population and their difference of distribution between 2009, 2013 and 2017.

	2009		2013	2017	
	n (%)		n (%)	n (%)	P-value
**Age of the child**		**n = 8105**	**n = 8844**	**n = 11022**	<0.001
2–7 years		4541 (56)	4615 (57)	7017 (59)	
8–12 years		3564 (44)	4229 (43)	4005 (41)	
**Gender of the child**		**n = 8105**	**n = 8844**	**n = 11022**	0.252
Male		4165 (51)	4341 (51)	5716 (52)	
Female		3940 (49)	4503 (49)	5306 (48)	
**Ethnicity of the child**		**n = 7830**	**n = 8444**	**n = 9586**	<0.001
Caucasian		7092 (92)	7816 (92)	9109 (94)	
Non-Caucasian		738 (9)	628 (8)	477 (56)	
**Education level of parents**		**n = 7253**	**n = 8128**	**n = 10110**	<0.001
Low		2163 (30)	2012 (25)	2246 (22)	
Middle		3106 (43)	3638 (45)	4373 (43)	
High		1984 (27)	2478 (30)	3491 (35)	
**Parents’ perception of the child’s weight status**		**n = 7927**	**n = 8772**	**n = 10995**	<0.001
Not heavy Heavy Extremely heavy		7420 (94) 489 (6) 18 (<1)	8221 (94) 536 (6) 15 (<1)	10497 (95) 470 (5) 28 (<1)	
**Child’s weight status based on BMI**^a^		**n = 7451**	**n = 7828**	**-**	
Not overweight		6610 (86)	7064 (90)	-	
Overweight		663 (9)	612 (8)	-	
Obese		178 (2)	152 (2)	-	

^a^ The child’s BMI was calculated using the child’s weight and height as reported by the parents.

The missing numbers including the percentages were, in 2009-2013-2017, respectively: Ethnicity of the child: n = 275 (3%), n = 400 (5%0, n = 1436 (13%); Education level of parents: n = 852 (11%), n = 716 (8%), n = 912 (8%); Parents’ perception of the child’s weight status: n = 178 (2%), n = 72 (1%), n = 27 (0,2%); Child’s weight status based on BMI: n = 654 (8%), n = 1016 (11%).

### Agreement between the parents’ perception of their child’s weight status and the child’s actual weight status based on BMI

The percent agreement between the parents’ perception of their child’s weight status and the child’s actual weight status are summarized in Table 2. The concordance (the proportion of those with fully agreement) in 2009 was 89.7% and in 2013 91.5%. Note that the percent level of agreement differs between the various categories of weight status. Specifically, among the children who were classified as “overweight” according to their BMI data, only 34.6% and 38.4% of their parents considered their child to be “heavy” in 2009 and 2013, respectively. On the other hand, 64.7% and 61.0% of parents in 2009 and 2013, respectively, underestimated the weight status of their overweight child, reporting that they perceived their child as not heavy. Strikingly, in 2009 and 2013, 95.5% and 93.0% of parents, respectively, underestimated the weight status of their obese child, reporting that they perceived their child as either heavy (36.2% and 38.3% in 2009 and 2013, respectively) or not heavy (59.3% and 54.7% in 2009 and 2013, respectively).

**Table 2:** Percent agreement between the child’s actual weight status based on BMI and the parents’ perception of their child’s weight status and Cohen’s kappa values.

	Weight status based on BMI^a^			Total
	Not overweight n (%)	Overweight n (%)	Obese n (%)	n (%)
**Parents’ perception in 2009**				
Not heavy	6423 (97.5)	425 (64.7)	100 (59.3)	**6948 (93.6)**
Heavy	172 (2.5)	229 (34.6)	64 (36.2	**465 (6.2**
Extremely heavy	0 (0.0)	6 (0.7)	12 (4.5)	**18 (0.2)**
**Total**	**6595 (100)**	**660 (100)**	**176 (100)**	**7431 (100)**
**Parents’ perception in 2013**				
Not heavy	6877 (97.9)	352 (61.0)	85 (54.7)	**7314 (94.2)**
Heavy	164 (2.1)	251 (38.4)	58 (38.3)	**473 (5.6)**
Extremely heavy	1 (0.0)	3 (0.6)	8 (7.0)	**12 (0.2)**
**Total**	**7042 (100)**	**606 (100)**	**151 (100)**	**7799 (100)**

Complex sample analysis was performed because the dataset incorporated a complex sampling design (unequal sampling) and a sample weight was assigned to each person sampled.

^a^ The child’s BMI was calculated using the child’s weight and height as reported by the parents

In 2009 and 2013, Cohen’s kappa was 0.38 (95% CI 0.35–0.41), indicating a moderate agreement and 0.43 (95% CI 0.40–0.46), indicating a reasonable agreement, respectively. The difference between the observed kappa in 2009 and 2013 was significant (Z = 2.0, p<0.05).

### Role of BMI and demographic factors on parents’ perception of their child’s weight status

Our analysis revealed that the child’s actual weight status affected the parents’ perception of their child’s weight status; thus, overweight and obese children are more likely to be assessed as “heavy” or “extremely heavy” (with an odds ratio of 22.8 and 31.0 in 2009 and 2013, respectively) (Table 3). In addition, socio-demographic characteristics also influenced parents’ perception of their child’s weight status. In both 2009 and 2013, parents of children 2–7 years of age and parents of boys are less likely to estimate their child’s weight status as “heavy” or “extremely heavy”, regardless of the child’s actual weight status. Moreover, in 2009, parents with a middle or high education level were also less likely to estimate their child’s weight status as “heavy” or “extremely heavy”, regardless of the child’s actual weight status. In contrast, the child’s ethnicity had no effect on the parents’ perception of his/her weight status. These data are summarized in Table 3. No significant interactions were identified between the demographic factors and the child’s actual weight status based on BMI. The results of model 1 were very similar to the fully adjusted results of model 2; all variables that had a significant association in model 1 also had a significant association in model 2.

**Table 3:** Role of weight status and demographic factors on parents’ perception of their child’s weight status (logistic regression analyses).

	2009 Model 1^b^			Model 2. Fully adjusted^c^			2013 Model 1^b^			Model 2. Fully adjusted^c^		
	n	OR (95% CI)	P-value	n	OR (95% CI)#	P-value	n	OR (95% CI)	P-value	n	OR (95% CI)#	P-value
**Weight status child**^a^:	**7431**			**6795**			**7799**			**7325**		
not overweight		ref			ref			ref			ref	
overweight/obese		22.8* (17.5–29.6)	<0.001		27.8* (20.5–37.5)	<0.001		31.0* (24.5–39.2)	<0.001		40.0* (30.6–52.4)	<0.001
**Child’s age:**	**7431**			**6795**			**7799**			**7325**		
8–12 years		ref			ref			ref			ref	
2–7 years		0.19* (0.14–0.26)	<0.001		0.21* (0.15–0.29)	<0.001		0.22* (0.17–0.29)	<0.001		0.21* (0.16–0.28)	<0.001
**Child’s gender:**	**7431**			**6795**			**7799**			**7325**		
female		ref			ref			ref			ref	
male		0.61* (0.47–0.80)	<0.001		0.63* (0.47–0.84)	0.002		0.76* (0.60–0.96)	0.018		0.70* (0.54–0.90)	0.005
**Child’s ethnicity:**	**7290**			**6795**			**7582**			**7325**		
Non-Caucasian		ref			ref			ref			ref	
Caucasian		0.81 (0.53–1.22)	0.308		0.73 (0.43–1.25)	0.249		1.33 (0.88–2.01)	0.175		1.59 (0.97–2.60)	0.069
**Education level of parents:**	**6799**			**6795**			**7327**			**7325**		
low		ref	<0.001		ref	0.011		ref	0.366		ref	0.609
middle		0.58* (0.42–0.79)			0.67* (0.48–0.93)			0.96 (0.73–1.27)			1.13 (0.84–1.53)	
high		0.47* (0.32–0.68)			0.59* (0.40–0.88)			0.79 (0.57–1.11)			0.98 (0.69–1.39)	

The dependent variable parental perception of their child’s weight status, 0 = not heavy, 1 = heavy or extremely heavy.

OR, odds ratio; CI, confidence interval

^a^ The child’s BMI was calculated using the child’s weight and height as reported by the parents

^b^ In model 1: the effect of each demographic variable was corrected for the child’s weight status based on BMI

^c^ Model 2 was adjusted for the child’s weight status based on BMI and all demographic variables simultaneously

*p<0.05.

### Role of socio-demographic factors on parents’ underestimation of their child’s weight status among overweight and obese children

In 2009, the following groups of parents were more likely to underestimate their overweight/obese child’s weight status: parents with a younger child (2–7 years of age; OR = 0.18, 95% CI 0.12–0.26); parents of a boy (OR = 0.55, 95% CI 0.39–0.79); and parents with a middle and high level of education (OR = 0.56*, 95% CI 0.36–0.85) and (OR = 0.44, 95% CI 0.26–0.77) respectively). In 2013, only parents with a younger child (2–7 years of age) were more likely to underestimate their child’s weight status (OR = 0.22, 95% CI 0.16–0.31) and parents with a high level of education (OR = 0.59, 95% CI 0.40–0.91). These data are presented in Table 4. We also analyzed for both years together and found that the interaction between education level of parents and years is significant (p<0.001). For that reason we decide to present our results for both years separately.

**Table 4:** Role of demographic factors on parents’ perception of their child’s weight status of parents of overweight/obese children^a^ (logistic regression analyses).

	2009			2013		
	n	OR (95% CI)	P-value	n	OR (95% CI)	P-value
**Child’s age:**	**836**			**757**		
8–12 years^b^		ref			ref	
2–7 years^b^		0.18* (0.12–0.26)	<0.001		0.22* (0.16–0.31)	<0.001
**Child’s gender:**	**836**			**757**		
female^b^		ref			ref	
male^b^		0.55* (0.39–0.79)	0.001		0.81 (0.60–1.10)	0.175
**Child’s ethnicity:**	**811**			**726**		
Non-Caucasian^b^		ref			ref	
Caucasian^b^		0.90 (0.54–1.51)	0.689		1.34 (0.82–2.18)	0.249
**Education level of parents:**	**724**			**683**		
low^b^		ref	0.003		ref	0.044
middle^b^		0.56* (0.36–0.85)			0.96 (0.67–1.38)	
high^b^		0.44* (0.26–0.77)			0.59* (0.40–0.91)	

The dependent variable parental perception of their child’s weight status, 0 = not heavy, 1 = heavy or extremely heavy.

OR, odds ratio; CI, confidence interval

^a^ The child’s weight status was calculated using the child’s weight and height as reported by the parents.

^b^ Corrected for the child’s weight status based on BMI

*p<0.05.

## Discussion

The aim of this study was to examine the level of agreement between the parents’ perception and the child’s actual weight status, the factors that moderate this perception, and changes over time in a large Dutch cohort of parents of children 2–12 years of age. This level of agreement was higher in 2013 compared to 2009.

In 2013, fewer parents underestimated their child’s weight status compared to 2009. However, in both years, the kappa value for agreement between the parents’ perception of their child’s weight status and the child’s actual weight status based on BMI was lower than the Kappa value for a good agreement as defined in the Methods section [32, 34]. Therefore, we conclude that the inter-method reliability is not satisfactory [35], and parents’ underestimation of their child’s weight status persisted in 2013.

The parents’ perception of their child’s weight status did not change significantly between 2009, 2013, and 2017, with approximately 5–6% of parents classifying their child’s weight status as “heavy” or “extremely heavy”. In addition, our data show that parents of overweight/obese children tend to underestimate their child’s weight status; two-thirds of parents of overweight children 2–12 years of age underestimated their child’s actual weight status in both 2009 and 2013. Among parents of obese children, 95.5% and 93.0% of parents underestimated their child’s actual weight status in 2009 and 2013, respectively, failing to classify their child’s weight status as “extremely heavy”.

Irrespective of the child’s actual weight status based on BMI, parents of children 2–7 years of age, parents of boys, and parents with a middle/high education level were significantly less likely to estimate their child's weight status as either heavy or extremely heavy. Despite this finding, the only factors that were related to underestimation of overweight/obese children’s weight status in both 2009 and 2013 were younger age (i.e., 2–7 years of age) and higher educational level of parents. The finding that parents tend to estimate their child’s weight status more accurately as their child ages is important; although overweight and obesity are prevented and treated more easily in younger children, this is more challenging because the parents’ underestimation of their child’s overweight/obesity status is associated with younger age. In addition. further research is needed to understand why higher educated parents are more likely to underestimate their child’s weight status.

### Strengths and limitations

We consider our large sample size of the population and the use of a validated questionnaire to collect data regarding the parents’ perceptions are particularly notable strengths, which contributes to the generalizability to similar populations. Another strength is that the parents who completed the Child Monitor questionnaire were unaware of the aim of our study. On the other hand, some limitations should be considered when interpreting our results. First, the child’s height and weight were not obtained by professionals with calibrated measuring instruments. This would have been preferred, but was practically not feasible. Thus, the calculated BMI that was potentially based upon the parents’ misperception or inaccurate home measurement of their child’s weight and height, may have affected our analysis of the parents’ estimation of their child’s weight status [36–39]. To test this possibility, we requested the height and weight values measured by Youth Health Care (YHC) professionals from the Municipal Health Services in the Gelderland-South region in 2009 and 2013 in children between 4–12 years of age. These measurements were taken during standard child's measurement moments by the YHC professionals in the Netherlands; our analysis revealed that the actual percentage of overweight/obese children 4–12 years of age in the Nijmegen region were slightly higher (13% in 2009 and 12% in 2013) than the results obtained in our study for children 4–12 years of age in the Nijmegen region (12% and 10%, respectively). This suggests that the calculated BMI, based on self-reported height and weight by the parents, were only slightly lower than the calculated BMI based on measured height and weight by the YHC-professionals, thereby increasing slightly the percentage of parental underestimation when it would have been compared to real measured data. This finding is consistent with findings reported by Akerman et al. [38], Brettschneider [39], and Timmermans [40], who reported that parents of overweight children generally underestimate their child’s BMI.

Another limitation of this study was that the 2017 Child Monitor questionnaire did not contain any questions regarding the child’s height or weight; therefore, we were unable to determine the child’s actual weight status in the 2017 dataset. However, we found that the parents’ perception of their child’s weight status did not change significantly between 2009, 2013, and 2017; approximately between 5 and 6% of the parents classifying their child’s weight status as heavy or extremely heavy. In 2017 the percentage of overweight children according the YHC of the GGD Gelderland-South region was 10.7%. Given the differences in the percentages of overweight children according to our dataset and the dataset of the YHC in the GGD Gelderland-South region in both 2009 and 2013, we expect there is still an underestimation of the child’s weight status in 2017 and so a constant underestimation of the weight status by parents of overweight and obese children over time. Another potential limitation of our study was the possibility of selection bias, given that 9% of parents did not provide their child’s height and/or weight when completing the 2009 and 2013 questionnaires and missing data was not imputed. Unfortunately, the database we used did not keep track of the reason for non-participation. If the majority of these parents had children who were overweight or obese (or were perceived by their parents as being overweight or obese), this may have changed our analysis.

### Comparison with other literature

Our percentages of parents’ underestimation of their overweight children’s weight status are consistent with previous studies, which range from 50% to 85% [17, 20, 22, 24, 26]. Our finding that parental underestimation among overweight and obese children is higher in younger children is also consistent with previous studies [16–18, 20, 41–44]. Thus, parents might be reluctant to label their child as “overweight” due to societal pressure towards maintaining lower weight and/or the social stigma often attached to obesity [45]. It is also possible that some parents simply do not consider their child to be overweight, as the popular media often depicts overweight children using images of severely obese children, which may distort the parents’ understanding of what qualifies as “overweight” [46]; alternatively, because parents might not consider their child to be overweight because he/she engages in physical activity, is not teased about his/her size, and/or has no obvious health issues [47]. Furthermore, many parents believe that overweight children generally outgrow this condition [17]. In this respect, it’s interesting to note that the accuracy of parents’ estimating their child’s weight status increases with the age of the child.

Moreover, we found that the child’s gender (in the 2009 and 2013 dataset) and the parents’ education level (in the 2009 dataset) were associated with the parents’ perception of their child’s weight status. The gender-based difference in 2009 with respect to parents’ perception might be explained by the fact that parents often rationalize differences in weight status between girls and boys as a normal difference between the two sexes [16, 17]. In this context, these parents may also be influenced by our society’s higher desire for lower weight status in girls compared to boys. This bias can ultimately cause parents to accurately perceive an overweight daughter, while simultaneously misperceiving an overweight son as a precocious, non-underweight child [41]. Several other research groups also reported that parents of a boy are more likely to misclassify the weight of their child compared to parents of a girl [21, 41, 48]; in contrast, Maynard et al. reported that parents of a girl are more likely to misclassify their child’s weight status [44]. On the other hand, nine out of 17 studies reviewed by Towns and D'Auria [16] and Jansen and Brug [22] found that gender did not play a role in the parents’ classification of their child’s weight status, which is consistent with the results of our 2013 dataset. Possible explanations for the discrepancies between studies may be due to differences in the age-range of included children, the BMI cut-off criteria to define overweight, reference frame for overweight, the size of the study population, and choices of conducted subgroup analyses [16, 17, 20]. In contrast to the findings in our 2009 dataset, several groups found that parents with low SES are more likely to underestimate their child’s weight status [25, 41, 43, 49], although Jansen and Brug reported that SES is not associated with the parent’s awareness of their child’s weight status [16, 22]. In contrast to these studies we found that the parents with a high level of education were more likely to underestimate their child’s weight status. Consistent with the findings reported by Towns and D'Auria [16] and by Jansen and Brug [22], we found no association between the child’s ethnic background and the parent’s awareness of their child’s weight status.

Thus, despite growing global awareness of the increasing rate of childhood overweight and despite a larger focus on weight in general (for example, through initiatives introduced at the local and national level), many parents are still unable to identify when their own child is overweight/obese [17].

### Implications with respect to practice, policy-makers, and future research

The findings reported here may help increase awareness among healthcare professionals by demonstrating that parents continue to underestimate their child’s weight status, particularly among parents of obese children 2–7 years of age, regardless of the child’s ethnicity and the parents’ education level. This is an alarming finding, given that both overweight and obesity are easier to prevent and reverse in younger children. Nevertheless, parents still need to be educated with respect to obesity in order to reduce misperceptions. Given the significant number of severe health conditions associated with childhood obesity, all healthcare professionals have an ethical obligation to delicately and proactively educate parents of an overweight child. Communication between healthcare professionals and parent forms the cornerstone of interventions designed to address childhood overweight and can form a sound basis for working together with overweight children and their families. In this respect, systematic population-based screening plays an important role, as it may provide regular opportunities to discuss the child’s weight status with the parents, thereby helping parents recognize their child’s true weight status. The child’s weight and height should therefore routinely be measured by a healthcare professional in youth health care services or in pediatric care. Because parents often underestimate their child’s weight status and because many parents do not take their child to a healthcare professional when their child is overweight preemptive measurements of the height and weight of all children is therefore useful. This will enable the healthcare professional to initiate a conversation with parents of children who are at risk of becoming overweight. Emerging evidence suggests that simply raising awareness may have beneficial effects; when parents are given a simple report outlining their child’s BMI, weight classification (underweight, normal weight, overweight, or obese), and general guidelines regarding diet and physical activity, they are able to assessing their child’s weight status more accurately [50]. Moreover, as mentioned above, when the parents’ perception of their child’s weight status is corrected, they are more likely to move from the precontemplation stage of change to the preparation or action stage of change their child’s lifestyle [18].

Although achieving accurate parental perception of the child’s weight status is not sufficient to fully prevent childhood overweight and obesity, it is an important first step. Even if parents accurately perceive the weight status of their overweight/obese child, they may still lack the motivation, skills, and parenting practices needed to manage their child’s weight status. Moreover, parents of an overweight/obese child may even employ counterproductive strategies such as extreme dietary restrictions and/or pushing their child into unhealthy energy balance‒related behaviors in an attempt to address their child’s weight status [51], which may actually result in a long-term increase in the child’s BMI. Thus, after achieving accurate parental perception of the child’s weight status, the logical next step is for parents to be given the skills needed to manage their child’s weight status, for example in the form of family-based interventions [52–54].

## Conclusions

In summary, despite increasing global awareness regarding the increasing rate of childhood overweight/obesity and a greater focus on weight in general, many parents are unable to recognize when their own child is overweight or obese [17]. Parents’ underestimation of their child’s weight status remains alarmingly high, particularly among parents of young, obese children. This underestimation is a clear barrier to preventing childhood overweight/obesity. All healthcare professionals should take this underestimation into consideration, support parents by regularly measuring the child’s height and weight and discuss the implications, and should actively encourage parents to take steps to prevent overweight/obesity in their children, when indicated.

## Supporting information

S1 Appendix (DOCX)

## Abbreviations

95% CI: 95% confidence interval
BMI: body mass index
IOTF: International Obesity Task Force
OR: odds ratio
SES: socio-economic status
YHC: Youth Health Care
