# Supplementary material for: Parents’ underestimation of their child’s weight status. Moderating factors and change over time: A cross-sectional study
Source: PLoS One. 2020 Jan 16;15(1):e0227761. doi: 10.1371/journal.pone.0227761 (PMC6964904; doi:10.1371/journal.pone.0227761)
Supplement: S1 Appendix — (DOCX) [file pone.0227761.s001.docx]

**S1 Appendix**: **Cut off points BMI recommended by the IOTF**

International cut off points for body mass index for overweight and obesity by sex between 2 and 18 years recommended by the International Obesity Task Force (IOTF) as described in table 4 of Cole, T.J., et al., Establishing a standard definition for child overweight and obesity worldwide: international survey. BMJ, 2000. 320(7244): p. 1240-3

| 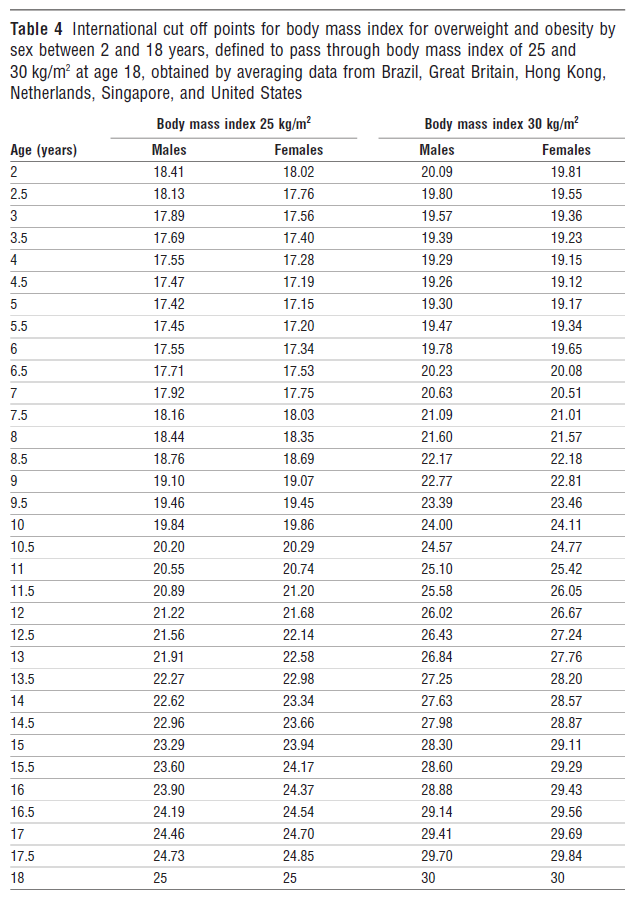 |
| --- |
